# Advanced Prodrug Strategies in Nucleoside and Non-Nucleoside Antiviral Agents: A Review of the Recent Five Years

**DOI:** 10.3390/molecules22101736

**Published:** 2017-10-16

**Authors:** Hanadi Sinokrot, Tasneem Smerat, Anas Najjar, Rafik Karaman

**Affiliations:** Department of Bioorganic & Pharmaceutical Chemistry, Faculty of Pharmacy, Al-Quds University, Jerusalem P.O. Box 20002, Palestine; hanadi12-10@hotmail.com (H.S.); asneem.toto@hotmail.com (T.S.); nash.najjar@gmail.com (A.N.)

**Keywords:** antiviral, macromolecules, prodrug, nucleoside, non-nucleoside, protide, targeted delivery

## Abstract

*Background*: Poor pharmacokinetic profiles and resistance are the main two drawbacks from which currently used antiviral agents suffer, thus make them excellent targets for research, especially in the presence of viral pandemics such as HIV and hepatitis C. *Methods*: The strategies employed in the studies covered in this review were sorted by the type of drug synthesized into ester prodrugs, targeted delivery prodrugs, macromolecular prodrugs, other nucleoside conjugates, and non-nucleoside drugs. *Results*: Utilizing the ester prodrug approach a novel isopropyl ester prodrug was found to be potent HIV integrase inhibitor. Further, employing the targeted delivery prodrug zanamivir and valine ester prodrug was made and shown a sole delivery of zanamivir. Additionally, VivaGel, a dendrimer macromolecular prodrug, was found to be very efficient and is now undergoing clinical trials. *Conclusions*: Of all the strategies employed (ester, targeted delivery, macromolecular, protides and nucleoside analogues, and non-nucleoside analogues prodrugs), the most promising are nucleoside analogues and macromolecular prodrugs. The macromolecular prodrug VivaGel works by two mechanisms: envelope mediated and receptor mediated disruption. Nucleotide analogues have witnessed productive era in the recent past few years. The era of non-interferon based treatment of hepatitis (through direct inhibitors of NS5A) has dawned.

## 1. Introduction

Despite the high efficiency of the human immune system, viruses are ubiquitous and versatile organisms with the potential to cause serious illnesses that require aggressive pharmacological intervention, yet existing medicines are by and large inefficient at combatting viruses, making them a target for aggressive exploration to accelerate the development of new antiviral agents.

The WHO estimates that there are a total of 36.7 million people living with HIV [1] and 257 million people living with hepatitis B [2]. These two main infections, as well as pandemics, have urged the scientific community to explore and develop novel and more potent antiviral agents.

There are two main approaches to antiviral prodrug design: the traditional or classical approach and the modern one. While the traditional approach aims to alter the physiochemical properties, such as lipophilicity by covalently modifying the drug either to increase its solubility by attaching it to hydrophilic functionalities [3] or to increase its passive permeability by attaching lipophilic moieties [4], the modern approach targets molecular and cellular entities such as membrane influx/efflux transporters and cellular protein expression and distribution [5].

In light of previously published articles which have reviewed antiviral drug strategies and novel antiviral agents [6,7,8,9,10,11,12], this review aims to explore strategies employed by researchers in the production of novel antiviral drugs and prodrugs published during the recent five years. The strategies were sorted by the type of drug synthesized into ester prodrugs, targeted delivery prodrugs, macromolecular prodrugs, other nucleoside conjugates, non-nucleoside drugs, and nanoparticles.

## 2. Ester Prodrugs

One of the more common and widely applicable prodrug design strategies is using an ester linkage to pair an existing active drug with an organic moiety. On the other side of the ester linkage, the chosen moiety has the potential to be another active agent, a carrier to exploit transfer mechanisms, or a lipophilic entity to increase permeability. Whichever the chosen moiety is, an ester linkage provides a readily cleavable site susceptible to both chemical and enzyme mediated cleavage.

**Figure 1:**
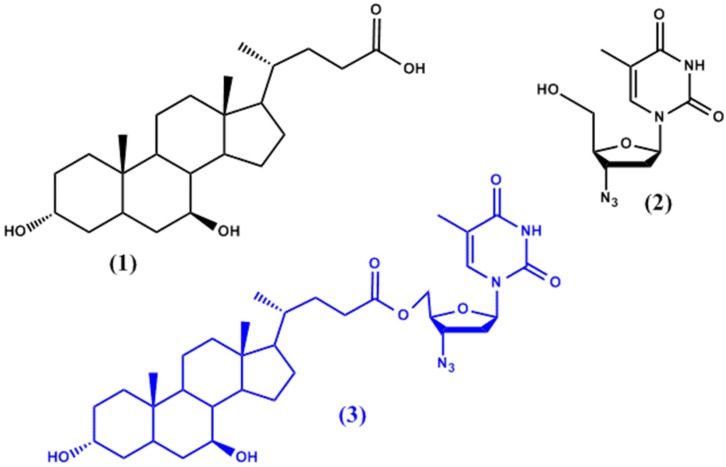
Chemical structures of ursodeoxycholic acid (UDCA) (**1**), azidothymidine (AZT) (**2**), and the prodrug (AZT-UDCA) (**3**).

Dalpiaz et al. [13] have attempted to use ursodeoxycholic acid (UDCA, **1** in Figure 1) to increase the permeability of azidothymidine (AZT, **2** in Figure 1) into the sanctuaries of HIV in the central nervous system (CNS) and macrophages. The resulting UDCA-AZT prodrug (**3** in Figure 1) (all prodrugs illustrated in the figures depicted throughout the review are highlighted in blue), which results from a 5′-ester conjugation of AZT with the bile acid ursodeoxycholic acid (UDCA), was found to be hydrolysed at slow rates in human plasma and whole blood (half-life: 7.5 h and 3.7 h, respectively) thus controlling AZT release. Moreover, it proved to be able to bypass active efflux CNS systems and to deliver up to twenty times more of AZT to the site of action in macrophages.

**Figure 2:**
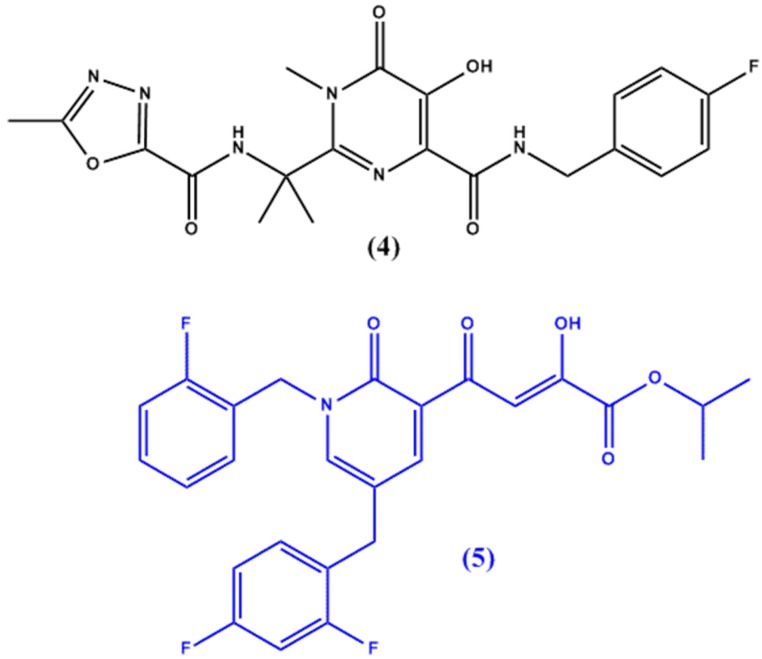
Chemical structures of raltegravir (**4**) and novel HIV integrase inhibitor **5**.

HIV-1 integrase, an enzyme required for HIV replication, is another target for anti-HIV agents. While raltegravir (**4** in Figure 2) is the only compound discovered to work on the HIV integrase enzyme, Seo et al. [14] have synthesized a lead compound, (4-(1.5-dibenzyl-1,2-dihydro-2-oxo-pyridin-3-yl)-2-hydroxy-4-oxobut-2-enoic acid), which was used in the design of potent HIV integrase inhibitors with favourable pharmacokinetic and pharmacodynamic profiles (in vitro half-life of >3 h post incubation in liver microsomes). The original lead compound suffered from poor cellular permeability which was improved by the synthesis of a novel isopropyl ester prodrug of a similar derivative (**5** in Figure 2).

## 3. Targeted Transporters Approach

Due to the main major drawbacks from which antivirals suffer, targeting intestinal transporters has great potential to facilitate their absorption. Hence, research exploiting this strategy has gained pace. Transporters targeted include organic anion transporters (OAT) [15], organic cation transporters (OCT) [16], sodium dependent bile acid transporters (ASBT) [17], sodium-dependent glucose transporters (SGLT) [18], monocarboxylate transporters (MCT) [19], and oligopeptide transporters (PEPT1) [20,21].

**Figure 3:**
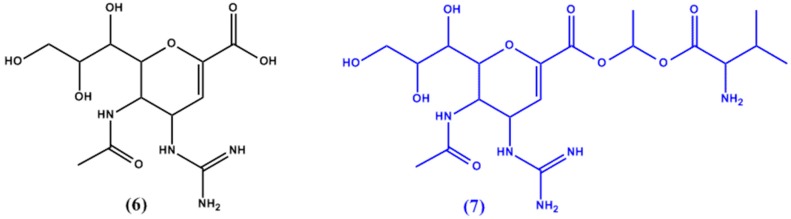
Chemical structures of zanamivir (**6**) and its valine ester prodrug (**7**).

Zanamivir’s (**6** in Figure 3) polar and zwitterion forms cause the drug to have poor bioavailability and render its use as a drug of choice for prodrug formulation [22]. Gupta et al. [23] thus aimed to target the intestinal transport pathway through PEPT1 by formulating a zanamivir and valine ester prodrug (**7** in Figure 3). The resulting prodrug exhibited substantially higher permeability (9 fold higher than zanamivir) and complete hydrolysis in mucosal cells, leading to the sole delivery of zanamivir.

**Figure 4:**
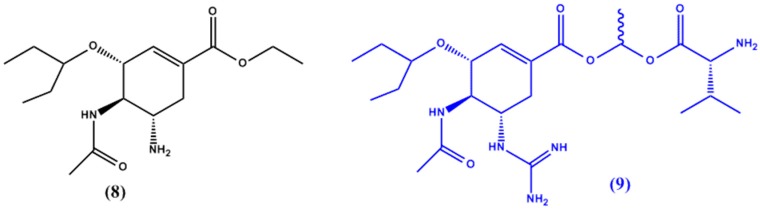
Chemical structures of oseltamivir (**8**) and its GOC-L-Val prodrug **9**.

In another study by Gupta et al. [24] a series of guanidine oseltamivir carboxylate (GOC) prodrugs has been synthesized to overcome the bioavailability drawbacks (high polarity) of the parent drug, oseltamivir (**8** in Figure 4). The prodrugs were also designed with PEPT1 targeting in mind. The prodrug with the ideal results was found to be GOC-L-Val (**9** in Figure 4); the valine ester of GOC, which showed enhanced absorption in mice when compared to the original drug.

**Figure 5:**
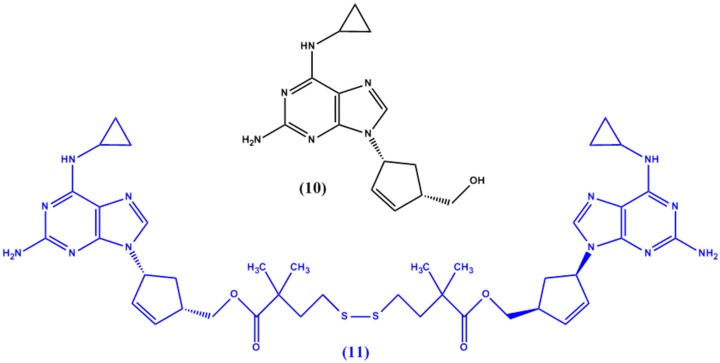
Chemical structures of abacavir (**10**) and its dimer prodrug **11**.

P-gp is an integral transport protein expressed extensively in intestinal epithelium as well as the blood brain barrier and is responsible for limiting the penetration of many drugs into the CNS. Anti-HIV drugs such as abacavir (**10** in Figure 5) have been shown to be good P-gp substrates when tested via in vitro experiments [25]. Namanja et al. [26] hence designed abacavir dimers to act as P-gp substrates. The idea behind the dimer entity is that it would act as a P-gp inhibitor and allow for high concentrations of the drug to entering the CNS while the intracellular environment would induce the cleavage of the dimer into the therapeutically active abacavir monomers [27]. The prodrug designed (**11** in Figure 5) was comprised of two abacavir molecules linked via a ‘traceless tether’—a molecule which would be removed completely from the monomers once inside the cells. This tether was a simple disulfide linkage linked via ester linkages at both ends to the abacavir monomers. The team showed that the dimer was a potent P-gp inhibitor and completely dissociates into monomers intracellularly.

## 4. Macromolecular Prodrugs

As a result of the high molecular weights of macromolecules (polymers) and their tunable potential, their application in the biomedical field has received considerable attention for the use as a tool for optimizing the pharmacokinetics profiles of conjugated drugs or proteins [28,29]. For instance, a significant number of studies have reported a vast value of the use of macromolecular-based prodrugs for improving the therapeutic activity of anticancer drugs [30,31,32,33].

As for antiviral drugs, the interest behind the applications of macromolecules is not solely limited to their potential in improving the delivery and pharmacokinetics of antiviral agents, but also due to the non-specific polymer-charge effect which is exhibited by macromolecules on viruses. Hence, leading to the inhibition of virus entry into mammalian cells [29,34]. Upon further investigation of the polymer charge effect, it became clear that poly-anionic macromolecules inhibit viruses extracellularly by denying them cell entry in the first place [35,36,37,38], while poly-cationic macromolecules, on the other hand, are complexed with the genetic material of the virus [39]. Moreover, macromolecules play an essential role in controlling the release, distribution, and nonselective accumulation of the conjugated drugs [40,41,42,43,44,45].

Macromolecule prodrugs strategy can be subdivided into natural, semi-synthetic, and synthetic macromolecule conjugates depending on the nature of the linkage between the parent drug and conjugate.

Previous research has been directed towards using naturally occurring macromolecules in the synthesis of antiviral prodrugs. One example is haemoglobin, which was used in an effort to improve the safety profile of ribavirin [46,47]. Furthermore, chondroitin sulphate, a sulphated natural glycosaminoglycan, was shown to have inhibitory action on dengue viruses through its interaction with envelop E protein resulting in diminished virus entry to cells [48].

However, natural macromolecules suffer from many drawbacks such as batch-to-batch variations and immunogenic reactions. Hence, research has been more directed towards the use of semi and fully synthetic polymers.

**Figure 6:**
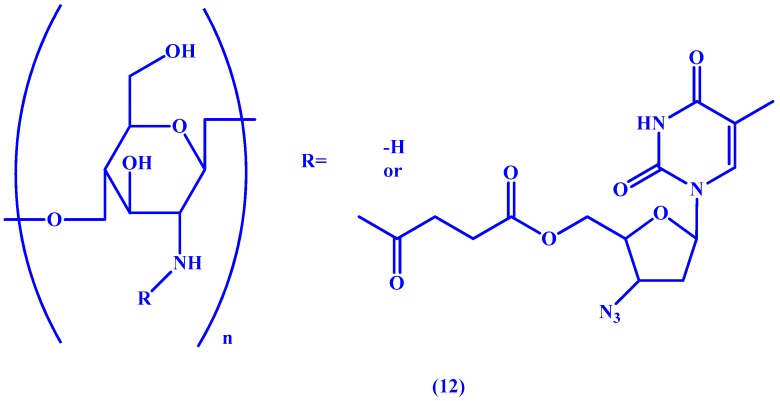
Chemical structure of the AZT-suc-COS prodrug **12**.

In a study by Liang et al. [49] chitosan (COS), a semi-synthetic polysaccharide formed from alkaline deacetylation of chitin, is used as a macromolecular carrier for AZT. In an effort to control the distribution of the drug and limit its rapid blood elimination, the linker of choice was a biodegradable succinic (suc) ester between AZT and the macromolecule which allows for sustained release of the drug (see **12** in Figure 6). The research team was able to confirm sustained release of the orally administered drug in mice as well as selective renal targeting via fluorescence imaging.

**Figure 7:**
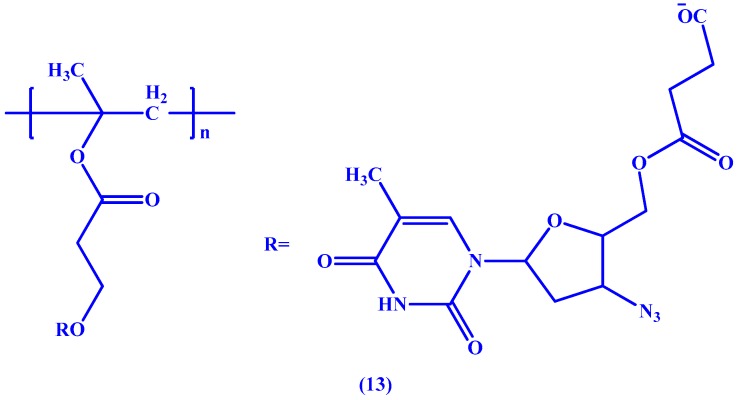
Chemical structure of the poly(HEMA)-AZT conjugate prodrug **13**.

Also employing the succinic ester linkage, Neeraj et al. [50] have conjugated AZT to poly-2-hydroxyethyl methacrylate (HPMA), a fully synthetic macromolecule, to obtain a macromolecular prodrug (**13** in Figure 7) with the aim of improving the pharmacokinetic profile of AZT. In vitro studies performed by the team showed that the resulting prodrug managed to increase both the half-life (8 h for the prodrug vs. 1 h for AZT) and bioavailability of AZT (1470 ng h/mL vs. 645 ng h/mL).

**Figure 8:**
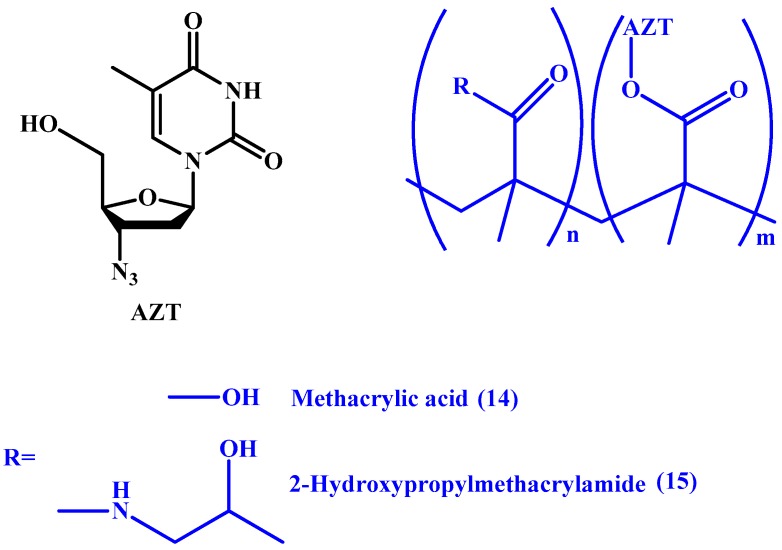
Chemical structures of PMAA-AZT (**14**) and HPMA-AZT (**15**).

Zuwala et al. [51], have also utilized acrylate derivatives in synthesizing two polymers. The group synthesized conjugates with poly(methacrylic acid) (PMAA) and HPMA (**14** and **15** in Figure 8, respectively), and claimed that the PMAA-AZT conjugate resulted in a longer duration of action when compared to the pristine drug. Furthermore, they reported that the conjugate exhibits its antiviral activity both extracellularly by adsorption and intracellularly on reverse transcription. However, the study was only able to visualize the internalization of the HPMA conjugate using fluorescence.

The major treatment-limiting side effects that ribavirin (RBV) causes is the uptake and accumulation in red blood cells which leads to inability to reach the site of action as well as results in haemolytic anaemia [52]. This offers ribavirin as a candidate of choice for the prodrugs approach.

**Figure 9:**
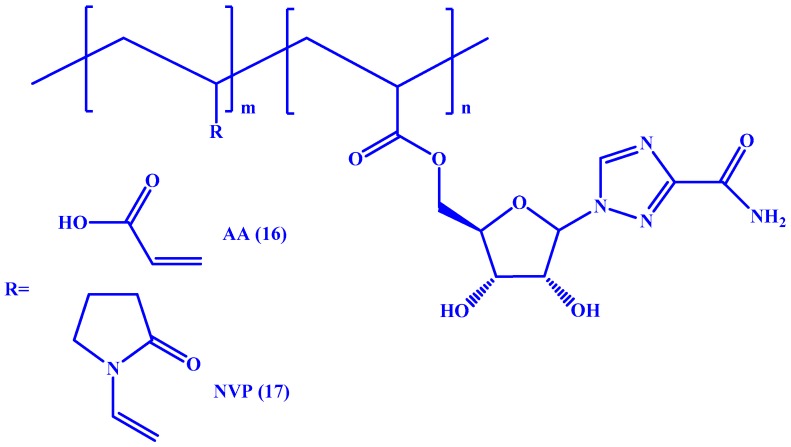
Chemical structures of RBV-AA (**16**) and RBV-NVP (**17**).

Kryger et al. documented the synthesis of two macromolecular prodrugs by polymerization of RBV-acrylate once with poly(acrylic acid) (AA) [53] and another with poly(*N*-vinylpyrrolidone) (NVP) [54] (see **16** and **17** in Figure 9, respectively). The resulting prodrugs showed no cytotoxicity to macrophages. Also, the poly(acrylic acid)-RBV conjugate exhibited decreased association with erythrocytes and high biocompatibility.

The macromolecular conjugate strategy is not limited to the use of linear macromolecules. In a study by Tyssen et al. [55], ‘dendrimers’, macromolecules comprised of a core with extensive well defined branching were studied. The core of the macromolecules was lysine functionalized with seven different functional groups being naphthyl and sulfonic acid derivatives. Two main factors play role in the potency and efficacy of synthesized dendrimers: (1) the size of the dendrimer and (2) the anionic surface charge of the dendrimer.

Of a series of dendrimers synthesized, SPL7013 was the best potential candidate as it exhibited broad-spectrum anti-HIV activity as well as long-lived protection against HSV-2 in mouse vaginal transmission model. SPL7013, also known as VivaGel^®^, exhibits its antiviral activity through blocking HIV-1 entry to the cells by blocking R5 and X4 envelope mediated cell-to-cell fusion as well as inhibiting recombinant HIV-1 reverse transcriptase. The authors report that VivaGel^®^, one of the dendrimers studied, was in clinical development. It is worth mentioning that VivaGel^®^ is now undergoing clinical trials [56].

## 5. ProTides and Nucleoside Analogues

Nucleoside-based antiviral agents are synthetic agents whose structure is similar to the naturally occurring nucleosides of DNA and RNA [12]. They are considered as the most common antiviral agents with immuno-modulating activity. However, nucleoside analogues suffered from undesirable pharmacokinetic profiles and poor antiviral activity leading to biological toxicity and drug resistance. Once a nucleoside analogue is taken up by the cell, it is activated via three intracellular phosphorylation steps through viral and cellular kinases to nucleoside-triphosphate, with the first phosphorylation of nucleosides representing the rate-limiting step.

In order to eliminate these drawbacks, previous work on formulating antiviral prodrugs which was focused on the synthesis and delivery of phosphorylated nucleoside analogues aiming to overcome the rate-limiting step. However, these analogues were faced by premature dephosphorylation and inefficient cellular uptake due to their charged nature leading to further research aimed towards improving them [57]. ProTides, a novel technology first employed by McGuigan [58], aims to mask the phosphoryl oxygen groups in order to mask their charge and improve cellular uptake. This strategy has proved successful in delivering drugs to the market such as sofosvubir and tenofovir alafenamide.

**Figure 10:**
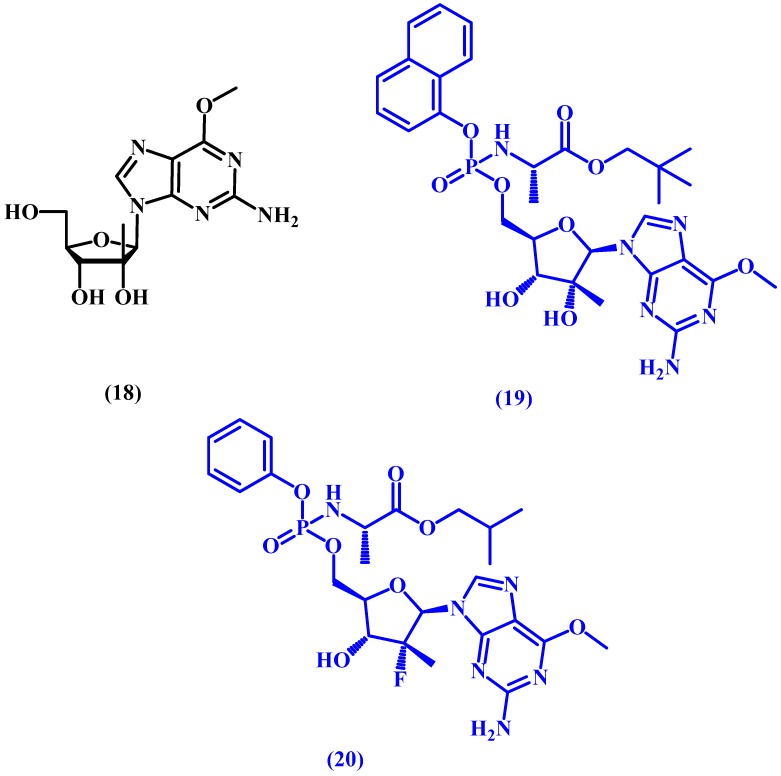
Chemical structure of guanosine derivative **18** and the prodrugs INX-08189 (**19**) and PSI-353661 (**20**).

INX-08189, also known as INX-189, is a potent hepatitis C (HCV) virus replication inhibitor prodrug prepared initially from a guanosine derivative (**18** in Figure 10). The starting compound undergoes masking and modification of the ester and amino acid moieties, as well as phosphorylation, which allows the drug to become active intracellularly. Afterwards, the prodrug is prepared by masking the phosphoryl group. INX-08189 (**19** in Figure 10) was found to be 500 times more potent than the parent nucleoside. Unfortunately, INX-08189 was discontinued in 2012 during phase II development due to its high cardiac toxicity [59,60,61].

Structurally similar to INX-08189, both PSI-353661 (**20** in Figure 10) and PSI-7977 have proceeded to clinical evaluation with impressive early results. PSI-353661, a protide prodrug, was discovered by Chang et al. in 2011 [62] and showed superior anti-HCV potency vs. the original guanosine derivative in addition to good stability (half-life > 24 h). PSI-7977 would later be acquired by Gilead Sciences under GS-7977 and eventually marketed as Sovaldi^®^.

**Figure 11:**
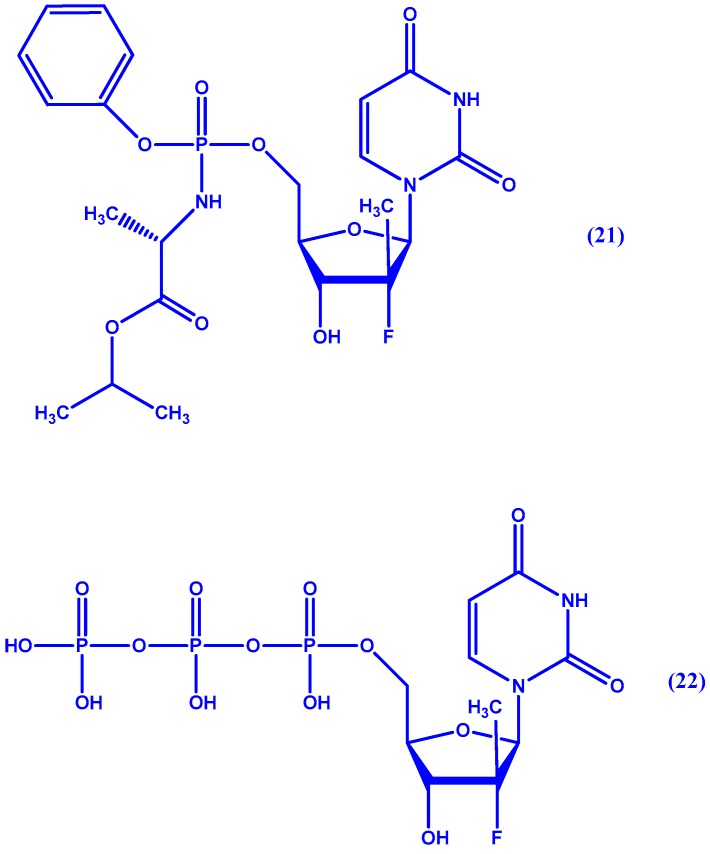
Chemical structure of sofosbuvir (**21**) and its active form **22**.

Sofosbuvir (**21** in Figure 11, formerly GS-7977), on the other hand, is a protide analogue which inhibits HCV NS5B polymerase and is now marketed under Sovaldi^®^ following successful clinical trials: PROTON, ELECTRON, QUANTUM, and ATOMIC [63]. Sofosbuvir is readily taken up by human hepatocytes and undergoes a series of metabolic steps both enzymatic and non-enzymatic. The final product is the triphosphate active form (see **22** in Figure 11) [64]. Though not a prodrug, ledipasvir (GS-5855) a potent direct inhibitor of HCV NS5A [65], was the product of work by Link et al. [66] done on a series of novel derivatives with benzimidazole-difluorene-imidazole cores. Following its success in clinical trials [67], it is now combined in a single oral tablet with sofosbuvir marketed under the name Harvoni^®^ by Gilead Sciences (Foster City, CA, USA).

**Figure 12:**
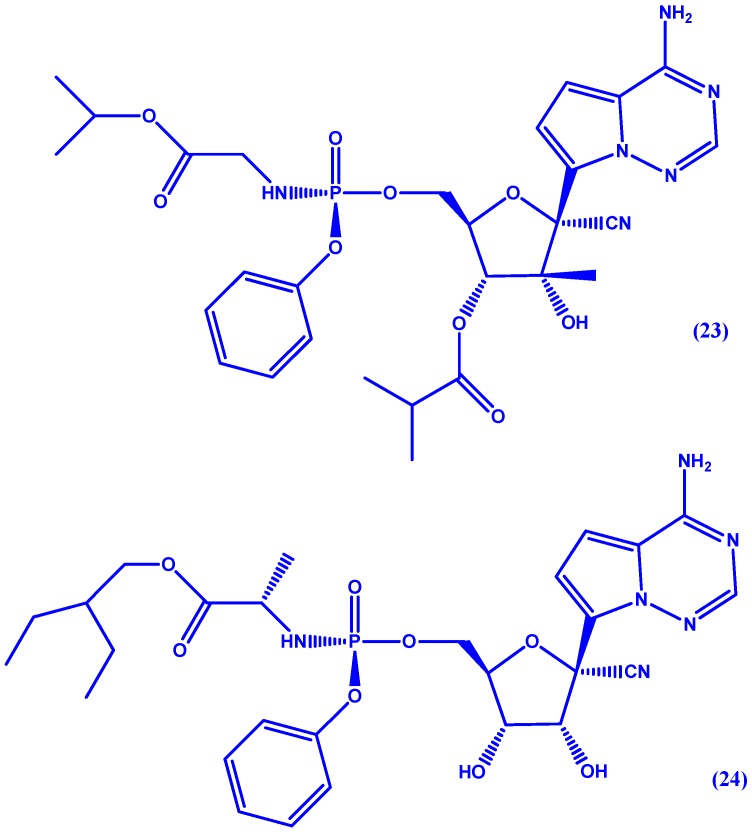
Chemical structure of GS-6620 (**23**) and GS-5734 (**24**).

GS-6620 (**23** in Figure 12), a C-nucleoside HCV inhibitor, was discovered by Cho et al. [68]. Named C-nucleoside for containing a 2′-C-methyl branched sugar it inhibits NS5B by preventing nucleoside-triphosphates from binding to the active site [69]. While this drug exhibited potency in phase I trials, it is hindered by intra- and inter-patient variability. Very recently, GS-5734 (**24** in Figure 12), has been discovered by Siegel et al. [70] for the treatment of Ebola virus (EBOV). The authors report pre-clinical efficacy in nonhuman primate EBOV challenge model (EC_50_ = 86 nM) with a Phase II study (PREVAIL IV) currently enrolling.

**Figure 13:**
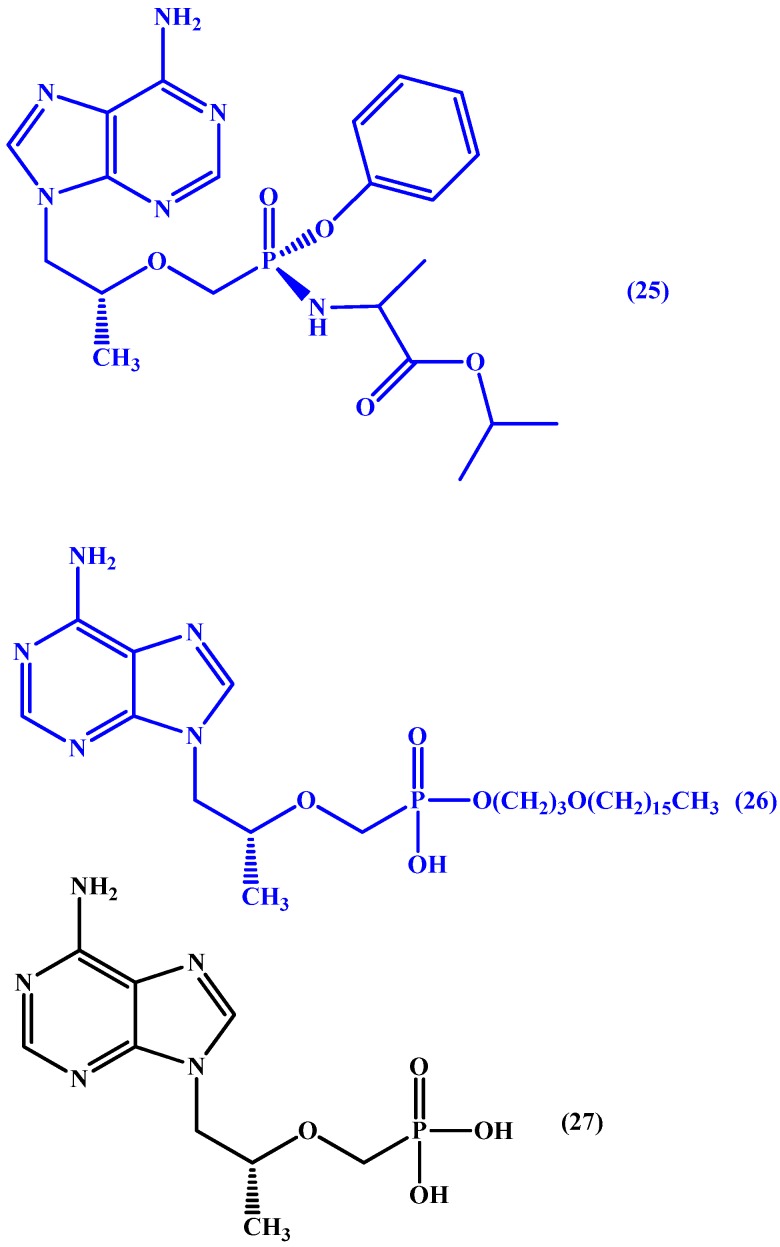
Chemical structures of tenofovir prodrugs, tenofovir alafenamide (**25**), CMX157 (**26**) and parent drug tenofovir (**27**).

Discovered in 2005 by Lee et al. [71], tenofovir alafenamide (GS-7340) (**25** in Figure 13), has been awarded FDA approval under the name Vemlidy^®^ for the treatment of hepatitis B. The prodrug is active orally and circulates in the plasma inactivated allowing for better distribution of tenofovir to tissues [72]. Also, it was found to be safe and well tolerated in a series of trials [73,74,75].

Also, CMX157 (**26** in Figure 13) a hexadecyloxypropyl conjugate of the acyclic nucleotide analogue of tenofovir (**27** in Figure 13). In phase I trials, the conjugate was found to have HIV hepatic targeting and was 267-fold more active than tenofovir against HIV-1 and 300 fold more active than tenofovir against multiple viruses in several different cell systems. Moreover, it was found to have wide activity against all major subtypes of HIV-1 and HIV-2 in fresh human peripheral blood mononuclear cells. Also, it was associated with higher intracellular levels of tenofovir–diphosphate conjugate than observed with tenofovir. The authors report that the increased antiviral activity can be attributed to increased cellular uptake and conversion to active diphosphate intracellularly. Moreover, a noticeable increase in CMX157’s selectivity index was reported due to decreased EC_50_ values in MT-2 cells and peripheral blood mononuclear cells (PMBCs) [61,76].

## 6. Non-Nucleoside Antiviral Agents

In addition to synthesizing nucleoside prodrugs to overcome the drawbacks mentioned above, another approach to designing novel antiviral agents is antiviral agents which are not based on nucleosides at all. This strategy aims to overcome the antiviral resistance towards nucleoside-based antiviral agents as well as provide more potent and pharmacokinetically attractive agents.

**Figure 14:**
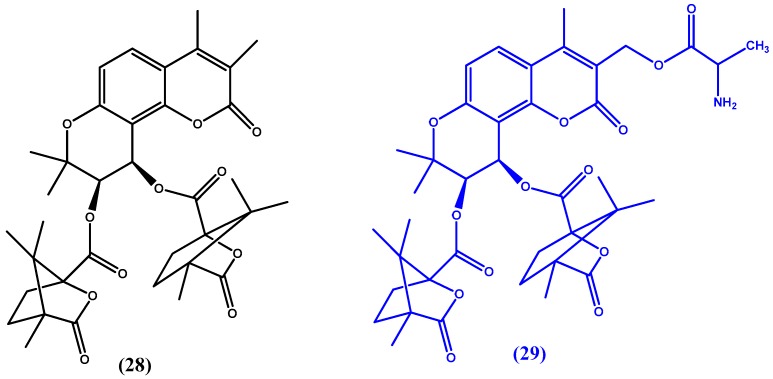
Chemical structures of 3′,4′-di-*O*-(S)-camphanoyl-(+)-*cis*-khellactone (DCK) (**28**) and [3′,4′-Di-O-(S)-camphanoyl-4-methyl-(+)-cis-kellactone-3-yl]methyl 2-aminopropanoate (**29**).

Guo et al. [77] continue their previous experimentation with khellactone derivatives, such as 3′,4′-di-*O*-(S)-camphanoyl-(+)-*cis*-khellactone (DCK) (**28** in Figure 14), [78] which has previously been identified as a potent anti-HIV agent acting through the inhibition of HIV-1 double-DNA strand formation [79,80]. The team investigated a series of camphanoyl-*cis*-kellactone molecules. Of the 13 synthesized and studied compounds ([3′,4′-Di-*O*-(S)-camphanoyl-4-methyl-(+)-*cis*-kellactone-3-yl] methyl 2-aminopropanoate) (**29** in Figure 14) showed the most promise in that it had the best pharmacokinetic profile and enhanced oral bioavailability when compared to the parent compound. The team believes that the molecule could be a potential anti-AIDS drug candidate.

**Figure 15:**
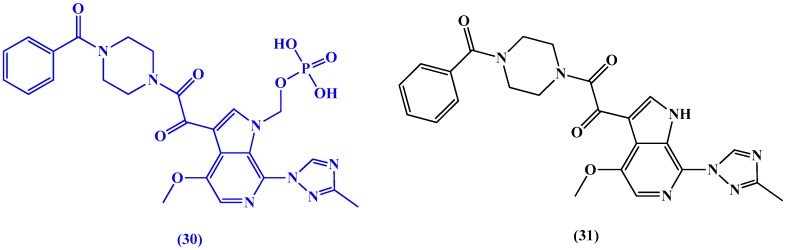
Chemical structures of the prodrug fostemsavir (BMS-663068) (**30**) and temsavir (**31**).

Regueiro-Ren et al. [81] have synthesized a series of HIV-1 attachment inhibitors derived from 4-fluoro-6-aza-indole analogues for clinical trials. The most promising candidate was fostemsavir (BMS-663068) (**30** in Figure 15), a prodrug of temsavir (**31** in Figure 15), which has reached phase III clinical trials [82]. The drug exhibits its HIV entry inhibitory action by blocking gp-120 HIV receptors hence preventing attachment.

**Figure 16:**
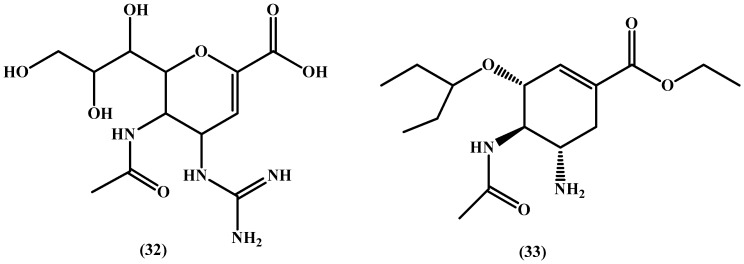
Chemical structures of zanamivir (**32**) and oseltamivir (**33**).

In a study by Schade et al. [83], a series of neuroaminidase inhibitor (NAI) prodrugs was synthesized with the structures of zanamivir and oseltamivir (**32** and **33** in Figure 16, respectively) in mind. The authors’ strategy was to improve the pharmacokinetics profile of zanamivir by decreasing its basicity and to overcome the emerging resistance to oseltamivir while maintaining its oral bioavailability. Taking inspiration from the potent 5-guanidino substituted oseltamivir, the team used *N*-hydroxylation and masking of polar groups as well as experimentation with *N*-hydroxyguanidine esters to identify potential prodrugs. Disappointingly, and keeping in mind the attractiveness of the strategies used by the authors, no convincing oral bioavailability was obtained, and hence, the bioavailability problems from which common NAIs suffer cannot be simply overcome by decreasing their basicity and solubility.

## 7. Nanoparticles

The modern approach to using nanoparticles as platforms for delivery of pharmaceutically active compounds is being widely studied. Literature on the use of nanoparticles as antivirals, such as gold and silver nanoparticles, has previously been reported [84,85,86,87]. Though, by loading active antivirals onto nanoparticles, researchers aim to achieve better pharmacokinetics as well as targeted delivery.

In a study by Russo et al. [88] foscarnet/chitosan nanoparticles were prepared. Foscarnet marketed under Foscavir^®^ is the trisodium salt of phosphonoformic acid. While it inhibits of herpesvirus DNA polymerase, drug- resistant cytomegalovirus (CMV) and HIV, it suffers from poor bioavailability and is administered intravenously. Through foscarnet induced gelation of chitosan, the resulting initial nanoparticle size was 200–300 nm increasing to 500–600 nm after being stable for 5 h in phosphate buffer saline. The authors report that due to mucoadhesion of chitosan to mucosal epithelium this delivery system could be effective for both oral and topical administration.

Cavalli et al. [89] report the use of β-cyclodextrin-poly(4-acryloylmorpholine) monoconjugate (β-CD-PACM) to formulate nanoparticles to deliver acyclovir. The resulting acyclovir-loaded nanoparticles were spherical and 200 nm in diameter. To measure the cellular accumulation of acyclovir, the authors tested for acyclovir concentrations in Vero cells and report superior delivery than the parent drug. Moreover, acyclovir-β-CD-PACM nanoparticles demonstrated higher potency than free acyclovir (IC_50_ 0.05 vs. 0.16).

In a study by Kumar et al. [90], lactoferrin nanoparticles were synthesized to improve pharmacokinetics of AZT. AZT was loaded into lactoferrin nanoparticles by sol-oil chemistry with the aid of olive oil leading to the encapsulation of AZT. The nanoparticles were spherical with a diameter of 50–60 nm and tested on rat. The authors report that the loaded nanoparticles show no organ related toxicity in contrast to free AZT. Also, as intended, longer circulation times were achieved due to increased half-life of the nanoparticles (3.07 in male rats/3.27 h in female rats vs. 1.75 in males/1.92 in females for free AZT).

## 8. Conclusions

Anti-viral agents hold plenty of potential for improvement due to the fact that they suffer from several drawbacks; mainly poor pharmacokinetics and development of resistance. Hence, the scientific community continually explores several strategies to overcome those drawbacks or synthesize more potent and attractive agents.

The strategies employed in the last years in anti-viral research can be categorized into simple or complex prodrugs. Of all the strategies employed: ester, targeted delivery, macromolecular, nucleoside and nucleotide analogues, non-nucleoside analogues, and nanoparticles, the most promising are macromolecular prodrugs; these explore the potential for conjugation of previously known drugs to natural, semi-synthetic, and synthetic macromolecules, and nucleoside analogues. Those macromolecules not only allow for the enhanced delivery of the anti-viral agent, but also for the macromolecule onto which the agent is attached to exert its own effect on the virus.

Among the successful macromolecules is chitosan (COS), which was found to be an efficient macromolecular carrier for AZT allowing for sustained release of the drug. Poly-2-hydroxyethyl methacrylate (HPMA) and poly(methacrylic acid) (PMAA) were also shown to be successful AZT carriers.

On the other hand, dendrimers have proven to be a very successful delivery system where the dendrimers VivaGel^®^ is now undergoing clinical trials. VivaGel works by two mechanisms: envelope mediated and receptor mediated disruption. Nucleotide analogues witnessed productive years in the recent past. Among successful examples of nucleotide analogues are sofosbuvir (formerly GS-7977), a nucleotide analogue which inhibits HCV NS5B polymerase and is now marketed under Sovaldi^®^ following successful clinical trials: PROTON, ELECTRON, QUANTUM, and ATOMI, and tenofovir alafenamide (GS-7340) which has been awarded FDA approval under the name Vemlidy^®^ for the treatment of hepatitis B. It can be said that the era of non-interferon based treatment of hepatitis (through direct inhibitors of NS5A) has dawned: Harvoni^®^ and Sovaldi^®^ are such examples.

Additionally, nanoparticles have been used to improve the pharmacokinetic properties of a number of antiviral agents: mucoadhesion of chitosan delivery system (foscarnet/chitosan nanoparticles) was found to be effective in the treatment of herpesvirus DNA polymeraseand HIV for both oral and topical administration. Further, acyclovir-β-CD-PACM nanoparticles demonstrated higher potency than free acyclovir (IC_50_ 0.05 vs. 0.16) to treat herpes simplex.
